# Neural correlates of ambient thermal sensation: An fMRI study

**DOI:** 10.1038/s41598-017-11802-z

**Published:** 2017-09-12

**Authors:** Hajime Oi, Teruo Hashimoto, Takayuki Nozawa, Akitake Kanno, Natasha Kawata, Kanan Hirano, Yuki Yamamoto, Motoaki Sugiura, Ryuta Kawashima

**Affiliations:** 1 Climate Control and Cooling System Engineering Group, Nissan Motor Co., Ltd., Okatsukoku 560-2, Atsugi, 243-0192 Japan; 2 Institute of Development, Aging and Cancer, Tohoku University, Seiryo-machi 4-1, Aoba-ku, Sendai 980-8575 Japan; 3 International Research Institute of Disaster Science, Tohoku University, Aoba, 6-6-4, Aoba, Sendai 980-8579 Japan

## Abstract

An increasing number of biometeorological and psychological studies have demonstrated the importance and complexity of the processes involved in environmental thermal perception in humans. However, extant functional imaging data on thermal perception have yet to fully reveal the neural mechanisms underlying these processes because most studies were performed using local thermal stimulation and did not dissociate thermal sensation from comfort. Thus, for the first time, the present study employed functional magnetic resonance imaging (fMRI) and manipulated ambient temperature during brain measurement to independently explore the neural correlates of thermal sensation and comfort. There were significant correlations between the sensation of a lower temperature and activation in the left dorsal posterior insula, putamen, amygdala, and bilateral retrosplenial cortices but no significant correlations were observed between brain activation and thermal comfort. The dorsal posterior insula corresponds to the phylogenetically new thermosensory cortex whereas the limbic structures (i.e., amygdala and retrosplenial cortex) and dorsal striatum may be associated with supramodal emotional representations and the behavioral motivation to obtain heat, respectively. The co-involvement of these phylogenetically new and old systems may explain the psychological processes underlying the flexible psychological and behavioral thermo-environmental adaptations that are unique to humans.

## Introduction

Thermal perception, including thermal sensations (i.e., hot, warm, cool, and cold) and thermal comfort (i.e., satisfaction, acceptability, and tolerance), involve physical and physiological processes as well as psychological processes. Research investigating the psychological processes associated with environmental thermal perception has increased in the last 15 years and, in the field of biometeorology, this attention parallels drastic changes in models of thermal perception. Previously, thermal comfort was synonymous with the neutrality of thermal sensation, which is determined by physical and physiological parameters^1^. Indoor experiments based on this type of static model produced a very narrow range of comfortable temperatures for humans and resulted in environmental standards that encouraged intensively air-conditioned buildings. However, in dynamic environments, such as the outdoors and naturally ventilated buildings, people are more tolerant of a wider range of temperatures^2–4^.

These findings prompted a shift from static to dynamic or adaptive models that require the inclusion of psychological processes. Moreover, this shift was strongly promoted by recent pressures to save energy in buildings due to global environmental concerns^5, 6^. The inclusion of psychological processes is also considered to be critical for a better understanding of the impact of the thermal environment on health and cognitive performance^7–9^. Human thermoregulatory behaviors, which include personal (e.g., removing an item of clothing), technological (e.g., turning on an air conditioner), and cultural (e.g., having a siesta in the heat of the day) responses, are also consequences of these psychological processes^10, 11^.

Recently, the number of studies investigating the psychological processes underlying environmental thermal perception has increased. Biometeorological surveys have demonstrated that both thermal sensation and thermal comfort are affected by a variety of psychological factors, including the landscape, expectations, agency with regard to thermal control, and sociocultural variables^12–15^. An experimental study showed that agency in thermal control moderates the sensory experience and improves the comfort of the room temperature^16^, while several social psychology experiments identified a mutual relationship between the perception of social warmth and environmental thermal perception^17–20^. Despite these findings, the relationship between thermal sensation and thermal comfort remains elusive^21^, which places further emphasis on the importance and complexity of the psychological processes involved in environmental thermal perception in humans. However, the contributions of these studies are indirect in terms of understanding the mechanisms underlying the psychological processes.

Functional neuroimaging has the potential to directly probe the psychological processes associated with environmental thermal perception. Over the last two decades, data regarding brain activation during innocuous thermal stimulation obtained by studies performed for a variety of reasons have accumulated. A significant amount of data has been obtained in an effort to map the central processing regions involved in noxious and innocuous thermal sensations^22–27^. Additionally, several studies have investigated the response characteristics of a specific cortical area, the insular cortex^28–30^, several have attempted to dissociate the neural correlates of conscious thermal perception from the actual physical temperature^31, 32^, and others have addressed the neural substrates of either thermoregulation^33–35^ or thermal comfort^36–38^. More recent studies have investigated the relationship between physical and social warmth^39, 40^. Of the 19 aforementioned studies, most reported activation of the insular (16 studies) and sensorimotor (10 studies) cortices during the administration of either high or low innocuous thermal stimulation. Additionally, activation of the basal ganglia and/or the posterior parietal, prefrontal, and anterior cingulate cortices were reported by more than one-third of these studies.

However, it is not clear whether these neuroimaging findings are sufficiently informative to understand the psychological processes of environmental thermal perception in two respects. First, in technical terms, the thermal manipulation employed in these studies seems quite different from our daily environmental thermal experience. It was primarily limited to the local skin surface of a certain body part and applied with a coin-sized contact apparatus or a handy thermal pack. Although four studies manipulated the temperature of a large body surface area by blowing cold air into a sleeping bag^36^ or using a water-perfused body suit^33–35^, the thermal experience of the subjects in these studies appears to be quite different from what is experienced in daily life. For example, these manipulations caused a drastic temperature change around the torso while the air around the head and the air breathed in remained unchanged, which is the opposite experience of daily life. Additionally, the results of these four studies were mostly inconsistent with one another and with the neuroimaging literature of thermal manipulation as a whole.

Second, in theoretical terms, the dominant models of neural responses used in these previous studies do not reflect the concepts of the two variables involved in environmental thermal perception. The neural correlates of the two variables should represent the mental thermometer and the comfort indicator, which predict the unidirectional neural responses for each of these variables. On the contrary, most previous studies have assumed a bidirectional response to thermal sensations (i.e., an increased response as the temperature deviates to higher or lower temperatures from a neutral skin temperature). Accordingly, a majority of these previous studies (12 studies) compared either high or low temperature alone against a neutral temperature. Furthermore, several used both high and low thermal manipulation and reported a bidirectional thermo-sensitive response in various regions^24, 25, 30, 33^. Two exceptions may be noteworthy: a unidirectional response to a decrease in temperature in the dorsal part of the posterior insula^28, 29, 35, 40^ and to an increase in temperature in the hypothalamus^33, 35^. In terms of thermal comfort, none of the previous studies dissociated its effects from those of thermal sensation. In the three studies that addressed thermal comfort, the subjects’ thermal comfort and discomfort were correlated with their sensation of high and low temperatures, respectively^36–38^. Although thermal comfort was dissociated from “sensation” in one study^37^, the latter referred to bidirectional “intensity” rather than a unidirectional high/low dimension. In addition, there was no overlap among the results of these three studies.

**Figure 1:**
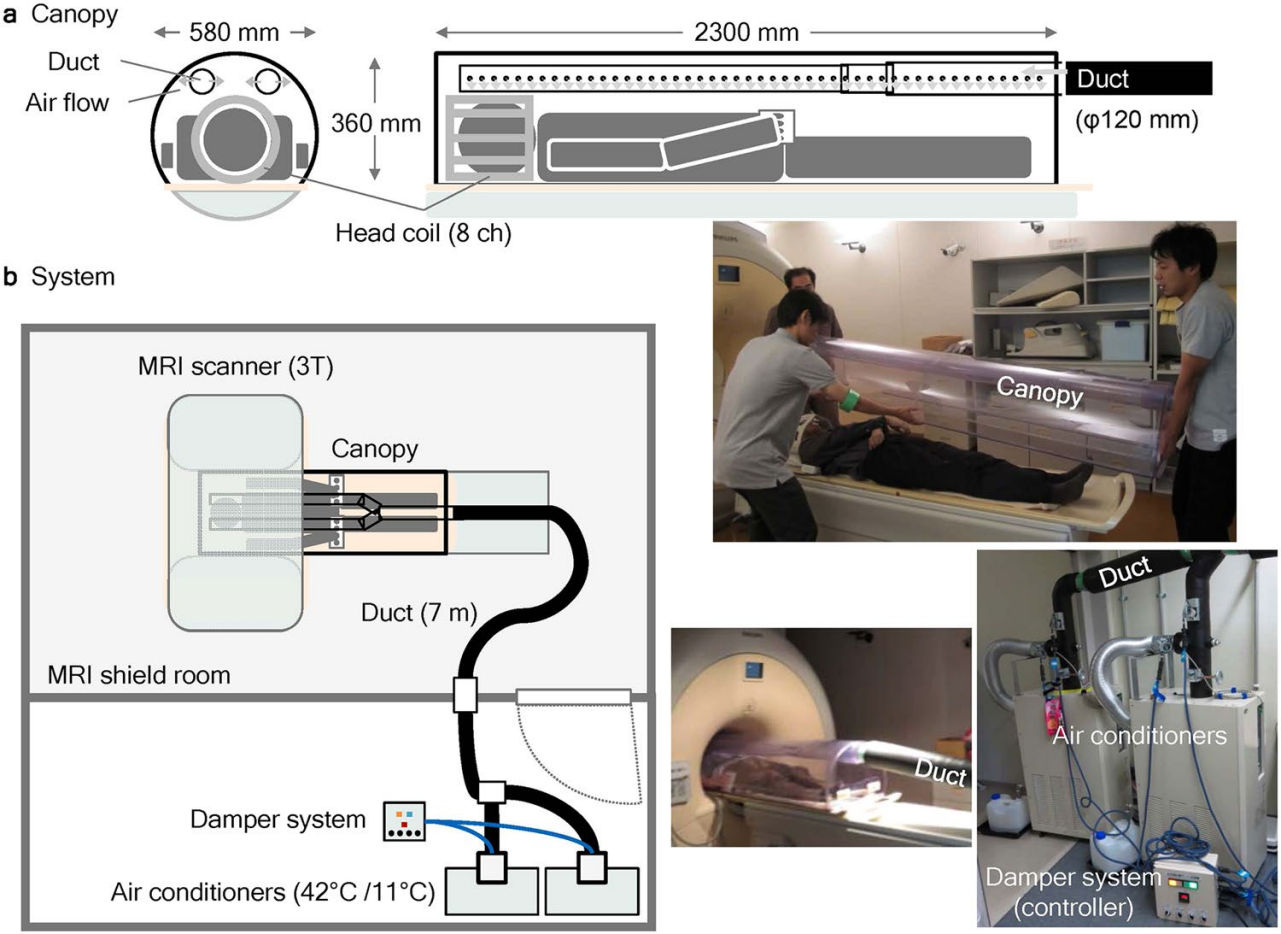
Equipment used for ambient thermal manipulation. (**a**) Each subject was covered with a custom-made canopy equipped with an inner duct system for air delivery that was made of clear vinyl chloride and fitted to the MRI gantry and bed. The inner duct system was connected with the external duct from the air conditioners at its foot-side end and bifurcated at the thigh level of the subjects to avoid the face and belly. The air blew out from the small side holes of the inner duct to avoid allowing the wind to directly hit the subjects. (**b**) Heating and cooling was controlled by air sent (3.3 m^3^/min) from one of two air conditioners (42 °C and 11 °C, respectively, at the outlet) placed outside the MRI scanner room. A damper system controlled the flow so that the air from one air conditioner went into the canopy through a flexible duct (φ 120 mm, approximately 7 m long). Due to heat loss and limited airflow speed, the effective range of the 10-min air temperature manipulation in the canopy was between 16–33 °C during the preliminary experiment.

**Figure 2:**
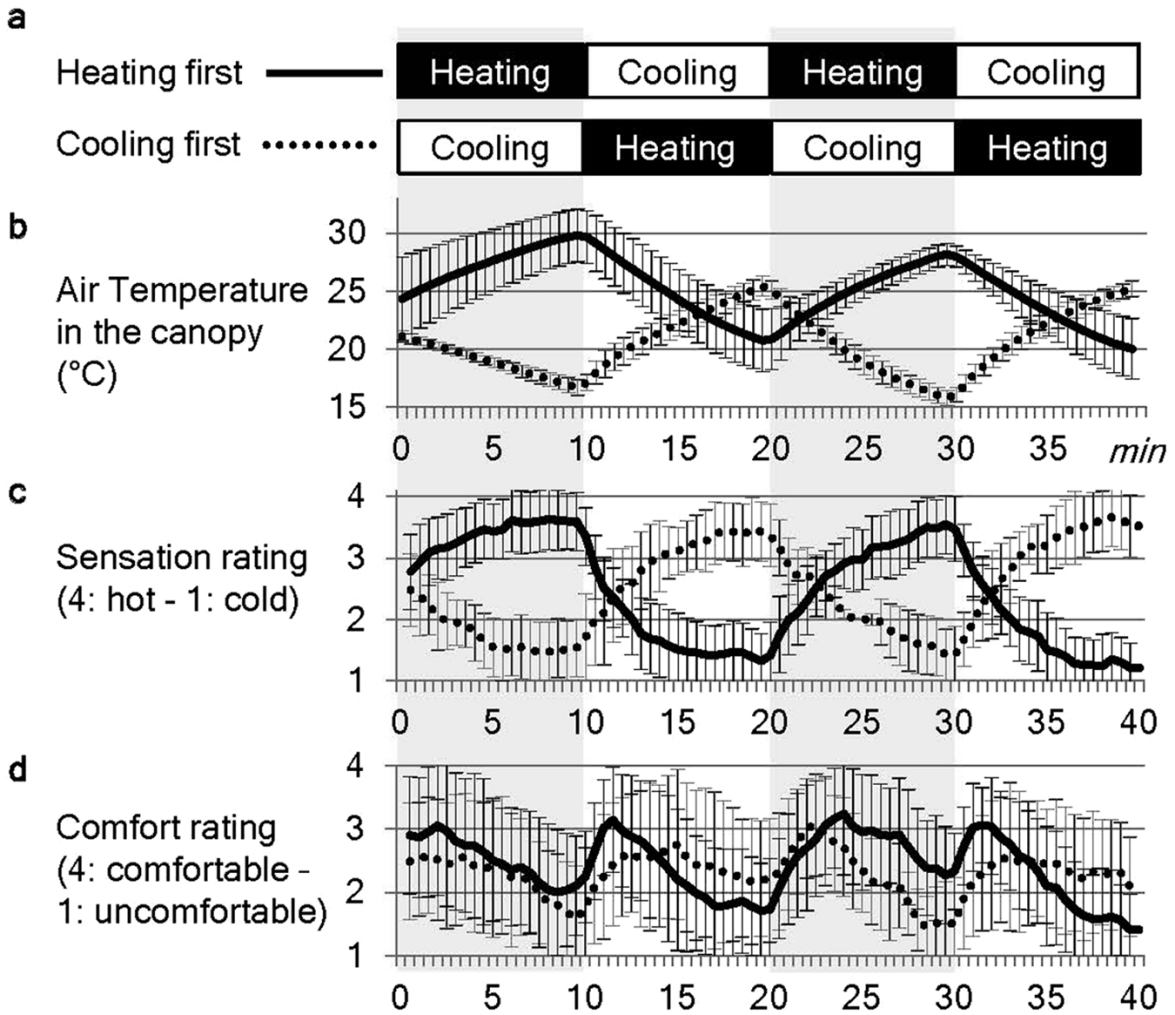
Thermal manipulation and perceptual measures. Each session consisted of two alternations of 10-min heating and 10-min cooling phases; the session began with the heating phase (heating first) for 18 subjects and with the cooling phase (cooling first) for 13 subjects (**a**). Corresponding average time-series data for the air temperature in the canopy (**b**) and two subjective measures (i.e., sensation and comfort ratings: (**c** and **d**) respectively) are shown separately for the heating first (solid line) and cooling first (dotted line) groups. Error bars indicate standard deviations.

Thus, the present functional magnetic resonance imaging (fMRI) study sought to identify the neural correlates of the psychological processes underlying naturalistic environmental thermal perception. To this end, ambient thermal manipulation and the dissociation of thermal sensation and comfort were included in a study paradigm for the first time. The subjects were covered with a large plastic canopy fitted to the size of the MRI gantry (Fig. 1a), and hot or cold air produced using an air conditioner located outside the scanner room was blown into the canopy through a duct (Fig. 1b). During the fMRI measurement, 10-min heating and 10-min cooling phases were alternated twice (Fig. 2a). Due to the limited speed of air flow and considerable amounts of heat loss from the duct and canopy, the inside-canopy temperature was expected to gradually increase and decrease, respectively, during each phase. All subjects were asked to independently rate their subjective levels of thermal sensation and comfort every 30 sec. The temperature change was intended to range between uncomfortably hot and cold with a comfortable range in between, which would cause the frequency of the time-series changes in thermal comfort ratings to be double those of the thermal sensation ratings; thus, the two ratings would be independent. These ratings were used for the regression analysis of the fMRI data to determine the neural correlates of thermal sensation and comfort, respectively.

## Results

### Thermal manipulation and perceptions

The present study included 31 healthy subjects; the session began with the heating phase (heating first) for 18 subjects and the cooling phase (cooling first) for 13 subjects (Fig. 2a). The experiment was performed during the winter (February–March). The air temperature in the canopy almost linearly increased and decreased during each of the heating and cooling phases, respectively (Fig. 2b). Data was not obtained from three subjects due to technical errors. Note that the variance in the temperature data may be attributed to differences in temperature in the daily room and/or the outside temperature or to the limited accuracy of the calibration of the MRI-compatible thermometer. The thermal sensation rating (hereafter referred to as “sensation rating”; Fig. 2c) generally changed in parallel with the inside-canopy temperature and exhibited apparent tendencies toward early acceleration and late deceleration within each 10-min manipulation period (i.e., alliesthesia). The thermal comfort rating (hereafter referred to as “comfort rating”; Fig. 2d) was associated with maximum discomfort at the hottest and coldest peaks of the sensation rating with the highest degree of comfort reported during the transient periods between these peaks. As expected, the frequency of the comfort rating time series (Fig. 2d) was double that of the sensation rating time series (Fig. 2c).

**Figure 3:**
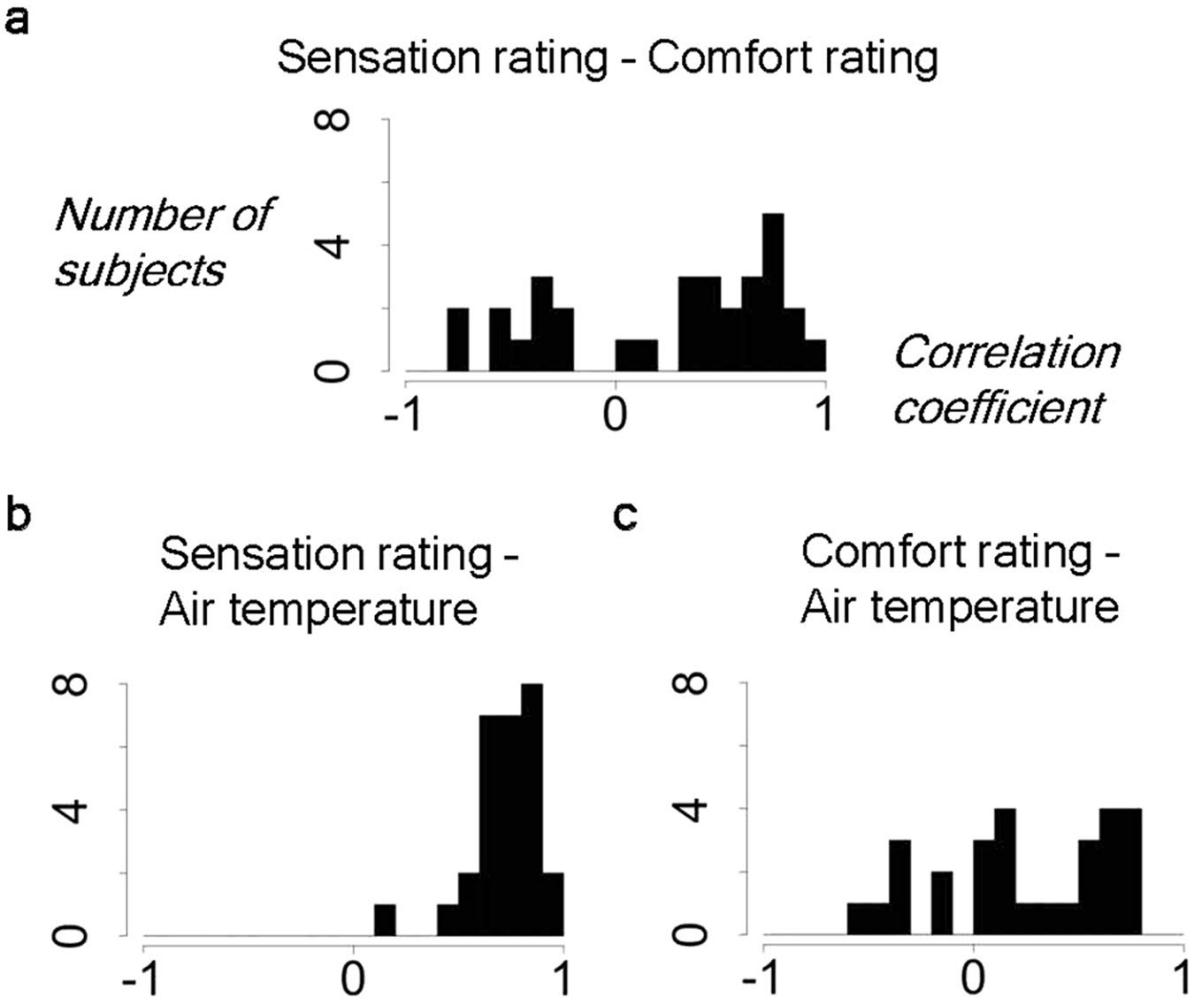
Within-subject correlations between the measures. Group distributions of the Pearson’s correlation coefficients between the two subjective measures (**a**) as well as between each subjective measure and the inside-canopy temperature (**b** and **c**).

The within-subject correlation between the sensation and comfort ratings was assessed to further determine their independence. In contrast to the expected orthogonal relationship from the averaged data (i.e., Fig. 2c and d), the group distribution of the between-ratings Pearson’s correlation coefficients (Fig. 3a) showed many positive (i.e., preferred hot) and negative (i.e., preferred cold) correlations. Furthermore, a one-sample *t*-test of the Z-transformed correlation coefficients revealed a significant dominance of the positive correlations (t_[30]_ = 2.593, *p* = 0.015 [two-tailed]), which suggests limited independence of the two subjective ratings and the need for a post-hoc examination of the fMRI data to determine a potential nuisance effect of this correlation. The correlations between each subjective rating and the inside-canopy temperature were also examined. The sensation rating had reasonably strong positive correlations (Fig. 3b; t_[27]_ = 16.324, *p* < 0.001) while the comfort rating showed both positive and negative correlations (Fig. 3c) with a significant positive bias (t_[27]_ = 3.293, *p* = 0.003), which is consistent with the positive between-rating correlation.

The respiration rate of each subject was recorded to eliminate the possibility of heat-induced hyperventilation, which causes a change in arterial CO_2_ pressure that produces artifacts in fMRI data. The respiration rate data of 23 subjects were collected; the data of eight subjects were missing due to technical errors. A similar analysis for the group distribution of the correlation coefficients did not reveal a significant correlation with either sensation rating (t_[22]_ = 0.171, *p* = 0.866) or comfort rating (t_[22]_ = 1.100, *p* = 0.283).

### fMRI data

A conventional two-level analysis of the multi-subject fMRI dataset was applied to the preprocessed images. At the individual level, the sensation and comfort ratings were included as regressors in the general linear model (GLM). The estimates for the sensation and comfort ratings were separately brought into the group-level one-sample *t*-test.

**Figure 4:**
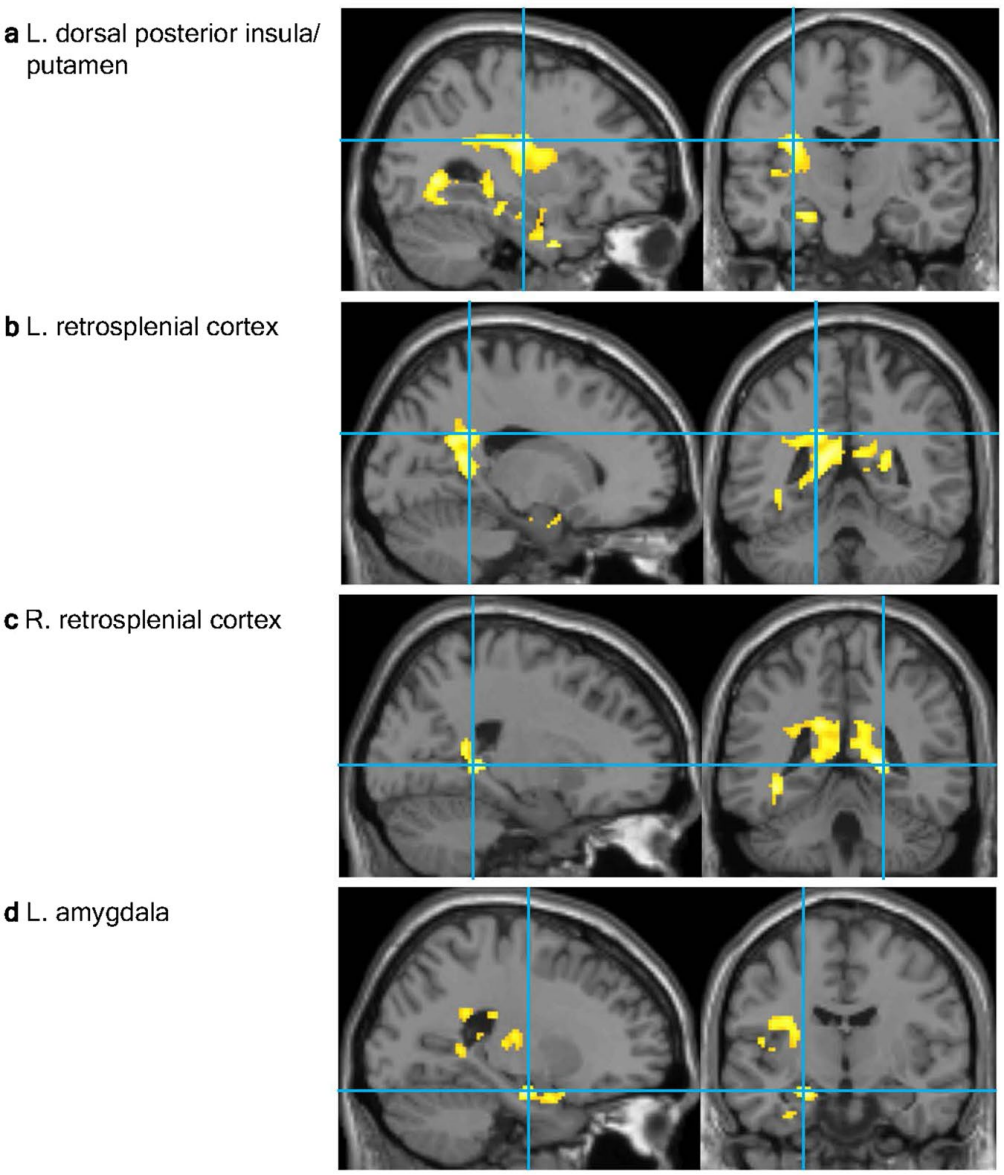
fMRI results. Activations correlated with the sensation of low temperature in the dorsal margin of the left posterior insula extending to the putamen (**a**), the left and right retrosplenial cortices ((**b** and **c**) respectively), and the left amygdala (**d**) presented using a red–yellow scale on the standard anatomical image of SPM12. The statistical threshold was set to *p* < 0.005 at the voxel level and corrected for a family-wise error (FWE) rate of *p* < 0.05 for multiple comparisons.

Three clusters exhibited a significant negative correlation with the sensation rating (i.e., activation for low temperature; Table 1). One cluster had peaks at the dorsal region of the left posterior insula (Fig. 4a) medially extending to the putamen, and at the left retrosplenial cortex (Fig. 4b); one cluster had a peak at the right retrosplenial cortex (Fig. 4c), and the other had a peak at the left amygdala (Fig. 4d). No significant positive associations with sensation ratings were identified, and no significant positive or negative associations with comfort ratings were identified.

**Table 1:** fMRI results.

Structure	L/R	MNI coordinates			*t*	k	*p* (*FWE*)
		x	y	z			
Positive correlation with sensation ratings (i.e., hot > cold)							
*n*.*s*.							
Negative correlation with sensation ratings (i.e., cold > hot)							
Insula (dorsal posterior)/Putamen	L	−30	−14	22	4.76	2028	<0.001
Retrosplenial cortex	L	−16	−46	28	4.30		
Retrosplenial cortex	R	22	−42	2	4.57	360	0.039
Amygdala	L	−22	−10	−18	4.62	557	0.004
Positive correlation with comfort ratings (i.e., comfortable > uncomfortable)							
*n*.*s*.							
Negative correlation with comfort rating (i.e., uncomfortable > comfortable)							
*n*.*s*.							

MNI coordinates (x, y, z) and t-values of the activation peaks, cluster size (k: number of voxels = 2 × 2 × 2 mm^3^) and the associated *p*-value (values corrected to family-wise error [FWE]) are presented for significant correlations of the sensation ratings or comfort ratings. *n*.*s*.: not significant.

Given the limited independence of the sensation and comfort ratings, whether or not the between-rating correlation in each subject influenced the identified activation for the low temperature was examined. A cross-subject correlation of the between-rating correlation coefficient (Z-transformed) and estimate for the sensation rating was not significant at any of the four peak voxels (*r* = 0.086, −0.043, −0.046, and 0.006, respectively; *p* = 0.647, 0.817, 0.805, and 0.972, respectively in the order of Table 1). Thus, the present findings indicate that there was no influence of the between-rating correlation.

Finally, to explore the potential gender difference, the estimates for the sensation and comfort ratings were separately brought into the group-level two-sample t-test. No significant difference was obtained.

## Discussion

The present fMRI study is the first to utilize ambient thermal manipulation and to investigate the neural correlates of thermal sensation and comfort separately. Significant associations were identified between brain activation and the sensation of low temperature in the left dorsal posterior insula, dorsal striatum, amygdala, and bilateral retrosplenial cortices.

The present findings regarding the left dorsal posterior insula are consistent with the idea that this region is the primary thermosensory cortex^28^. In monkeys, inputs from thermoreceptors in the skin and deep tissues to the spinal dorsal horn generate linearly graded responses to innocuous low temperatures in spinothalamic neurons that project to a specific contralateral thalamus relay nucleus. This thalamic nucleus projects to the fundus of the superior limiting sulcus at the dorsal margin of the middle/posterior insular cortex. The initial neuroimaging study of the human homologue of this cortical region utilized a small thermal stimulation apparatus^28^ and was followed up by additional studies that used a thermal pack^29, 40^ and a water-perfused suit^35^.

The present findings extended the notion that the left dorsal posterior insula is the primary thermosensory cortex to ambient environmental thermal sensation. The dorsal-most localization of the activated region in the posterior insula, extending to the adjacent parietal operculum, is worth noting. The idea of posterior insular unidirectional temperature coding has been challenged based on evidence that the posterior insula responds to warm sensations^33^, which has been previously reported^24, 30, 38, 39^. The unidirectional response is typically located dorsally, as shown in the peak coordinates z = 24^28^, 22 in the present study, 18^40^, 16–19^35^, and 14^29^, whereas the bidirectional peak is located ventrally with coordinates of z = −2–2^33^, 3^24^, 6^38^, 9^30^, and 16^39^. The apparent existence of more than two functional subregions in the posterior insula is consistent with recent views on the functional segregation of this region^41, 42^. On the other hand, there is no evident explanation for the left lateralization of the activations observed in the present study. Although bilateral activation was expected based on the principle of contralateral innervation, which is supported by studies using right hand stimulation^28, 29^, previous findings are inconsistent. For example, left-dominant activation was reported during body-suit warming^35^ whereas bilateral activation was reported during left hand stimulation^40^.

The activations of the retrosplenial cortex and amygdala are consistent with previous findings on whole-body thermal manipulation and may be relevant to supramodal emotional representations. For example, decreased activation of the retrosplenial cortex during hyperthermia was reported by a study using a water-perfused body suit^35^, and activation of the amygdala was observed during whole-body aerial-cooling in a sleeping bag^36^. Although the latter study interpreted the amygdalar activation in terms of the emotional response to thermal discomfort, the present findings dissociated the neural responses for sensation and comfort. The roles of the retrosplenial cortex and amygdala in the supramodal representation of emotions was recently suggested based on their activations during the perception of the emotional congruence between facial and vocal expressions (i.e., happy, angry, and neutral)^43^. However, the differential roles of the amygdala and retrosplenial cortex may be indicated by their early and late electroencephalogram responses, respectively, during pain perception^44^.

The involvement of the dorsal striatum may reflect the motivation for a behavioral adaptation to temperature (i.e., the subjects experienced a desire or urge to perform heat-seeking behaviors). This interpretation is common in rat studies following observations of increased metabolism in the dorsal striatum during shivering^45^ or the performance of operant lever-pressing to turn a heating lamp on and a cooling fan off^46^ to protect against environmental cooling. This dorsal striatal activity was replicated under conditions where the animal could not actually perform the behavior^46^ and the involvement of this region has also been observed in a hot environment^47^. The idea is congruent with the general role of the dorsal striatum in action selection and initiation through the integration of sensorimotor, cognitive, and motivational/emotional information^48^. In the present study, the activation was located in a lateral aspect of the left dorsal striatum, which is consistent with the involvement of the lateral dorsal striatum in stimulus-bound relatively automatic or habitual responses via connections with sensorimotor areas^48, 49^. Although dorsal striatal activation has been frequently identified during innocuous warming or cooling in previous neuroimaging studies, its meaning has rarely been discussed^23, 25, 27, 29, 30, 35^. One study speculated that the potential relevance of putamen activation during warming is related to reward and positive affect^38^, but this line of thinking is irrelevant to the present findings because the activation was independent of thermal comfort.

Thus, the results likely identified some components of a sophisticated neural mechanism that underlies the psychological processes associated with environmental thermal perception in humans. It may be relevant to note that the thermosensory cortex in the dorsal posterior insula is absent in sub-primates^50^. The representation of the physiological condition of the entire body in this cortical region is thought to be re-represented in the middle and anterior insula regions, which are areas found across hominoid primate species that act to integrate homeostatic information with sensory, hedonic, motivational, and social inputs^51^. The co-involvement of this phylogenetically new thermosensory system with the old systems for supramodal emotional representations (i.e., retrosplenial cortex and amygdala) and thermoregulatory motivation (i.e., dorsal striatum) may explain the apparently enigmatic psychological effects of environmental thermal perception in humans.

Thermal perception is modulated by a familiarity with^12^ or a preference for^15^ the environment. The retrosplenial cortex and amygdala, which are associated with place familiarity in the human brain^52^ and place preference in the rat brain^53^, respectively, may collaborate with the dorsal posterior insula to integrate place-relevant affective information with thermosensory input to produce these psychological effects in humans. Agency in action, which affects thermal perception when the action is relevant to thermal control^16^, and the perception of social warmth, which induces the perception of physical warmth^17–20^, involve co-activation of the insula and striatum^39, 54–56^. Additionally, environmental thermal perception and thermoregulatory behaviors are tightly connected with or restricted by the physical and social environments of humans. Thus, the co-functioning of these phylogenetically new and old systems may have evolved to enable humans to adaptively coordinate their perceptions and behaviors in complicated environments. This may explain why classical biometeorological models that account for only physical and physiological factors explain thermal perception under experimental conditions fairly well but are less successful in real dynamic environments.

The present study was unable to identify the neural correlates of thermal comfort. This was likely due to a lack of statistical sensitivity based on the limited speed of the thermal manipulation and the number of its repetitions. These features, which are shared with previous fMRI studies investigating environmental thermal perception^34, 36^, stem from two technical challenges. First, the limited speed of the thermal manipulation was due to the slow nature of the airflow and a considerable amount of heat loss. In the present study, the airflow speed was limited to minimize the subjects’ feeling of wind in the canopy and heat was lost primarily from the 7-m flexible duct and large canopy. Second, the number of repetitions of thermal manipulation (i.e., the length of the experimental session) was limited out of concern for the physiological load of the task on the subjects. It is rather invasive for the subject to remain lying quietly in a thermally-altered environment at uncomfortable temperature ranges for a long period of time. Although body temperature is essential for homeostasis, the subjects were not allowed to perform behavioral adaptations. These disadvantages related to statistical sensitivity may explain other negative findings as well, including the lack of heat-induced hypothalamic activation that has been reported previously^33, 35^. On the other hand, the number of subjects assessed in the present study (n = 31) was relatively large compared to those included in previous studies (eight^36^ and 20^34^), which somewhat compensates for the statistical disadvantage.

Three possible reasons for the failure to detect the neural correlates of thermal comfort remain. First, the thermal manipulation used in the present study might have been insufficient to produce significant levels of comfort or discomfort in the subjects. This possibility is supported by the fact that the average comfort ratings of the present subjects at the most comfortable and uncomfortable periods were only approximately 3 (slightly comfortable) and 2 (slightly uncomfortable), respectively (Fig. 2d). Second, each subject may have responded differently to the thermal comfort or discomfort at the neural level, which could have resulted in the absence of significant average activation. This possibility is in agreement with the variability of the adaptive response to thermal discomfort across individuals^10, 11^. Finally, it is possible that the experience of thermal comfort or discomfort does not share a neural response for high and low temperatures. This might explain why the neural findings of previous studies investigating thermal comfort or discomfort during cooling^36^, warming^38^, and a combination of these conditions^37^ do not converge at all. Future studies should address these three possibilities using new equipment and novel experimental designs.

Although the sensation and comfort ratings could not be perfectly orthogonalized, the differentiation of the neural correlates underlying thermal sensation from those supporting thermal comfort was considered to be successful. The tendency of the subjects in the present study to feel comfortable at a high temperature, as reflected by the significant dominance of positive correlations, may be explained by the fact that the experiment was conducted in winter. However, it is unlikely that this overall preference for the high temperature was influenced by activation for the low temperature sensation given the fact that there was no significant cross-subject correlation of the between-rating correlation coefficient and the estimate for the sensation rating at any of the activation peaks. One may suspect that the insufficient manipulation of comfort might have influenced the independence of the neural findings on thermal sensation from those of thermal comfort. However, this is unlikely. In the subjects who did not experience sufficient comfort or discomfort, related neural activation was simply not induced and, therefore, did not contaminate the analysis of thermal sensation.

Several methodological issues in the present study should be noted. First, rigorous control of the clothing worn by the subjects was not prioritized to create a casual mental set during participation in the experiment. While this was expected to contribute to the ability of the subjects to have a natural psychological response following environmental thermal changes, it might have resulted in an insufficient and variable manipulation. Second, the order and the length of the heating and cooling periods may not have been optimal. It is obvious that the air temperature range differed between the heating-first and cooling-first sessions (Fig. 2b). Finally, several measures that are known to affect thermal perception, including the physical information of the subject (e.g., height, body weight, body fat percentage, body surface area), temperatures of different body parts (i.e., surface and core), and humidity, were not obtained in the present study. Additionally, the measurement of air temperature in the canopy was not optimal due to thermal inhomogeneity in the canopy. The omission of these measures was based on consideration of the main interest of the present study, which was the subjective experience of each subject. Nonetheless, it is regretful that the absence of these measures prevents a comparison of the quality of the environmental thermal manipulation in the present study with those from the existing literature in the field of biometeorology.

## Conclusions

The present fMRI study aimed to determine the neural correlates of environmental thermal sensation and comfort during the manipulation of ambient temperature. Activation in the left dorsal posterior insula, dorsal striatum, amygdala, and bilateral retrosplenial cortices were correlated with the sensation of a low temperature. The activated region of the insula corresponded to the primary thermosensory cortex, which is considered to be a phylogenetically new structure that supports the mapping of bodily physiological information and its integration with other sensory, hedonic, motivational, and social information. The activation of the retrosplenial cortex and amygdala may be relevant to the supramodal emotional representation whereas the involvement of the dorsal striatum may reflect the motivation to engage in heat-seeking behavior that was automatically triggered by the thermal sensation. The co-involvement of these phylogenetically new and old structures may explain the psychological processes underlying the flexible psychological and behavioral thermo-environmental adaptations that are unique to humans.

## Methods

### Subjects

The present study included 31 healthy right-handed adults (16 males and 15 females) between 20 and 25 years of age (mean = 21.4 years). Subjects who presently had or reported a history of neurological and/or psychiatric diseases were excluded. Written informed consent was obtained from each subject, the experiment was conducted in accordance with the Declaration of Helsinki, and all procedures were approved by the Institutional Review Board of Tohoku University School of Medicine and the Nissan Motor Ethics Committee.

### Equipment

The equipment used in this study consisted of a custom-made canopy (Ohnishi Netsugaku Co., Ltd.; Tokyo, Japan) that covered the subject on the bed of the MRI scanner (Fig. 1a), two air conditioners (PAU-AZ1800SE, Apiste Corporation; Osaka, Japan), a custom-made damper system (Ohnishi Netsugaku Co., Ltd.), and a flexible duct (φ 120 mm, approximately 7 m long) that connected them (Fig. 1b). The canopy was made of clear vinyl chloride and was fitted to the size of the MRI gantry and bed (2,300 mm in length, 580 mm in width, and 360 mm in height). A duct system for the air delivery was built inside the canopy using the same material, connected to the external flexible duct (from the air conditioners) at its foot-side end, and bifurcated at the thigh level of the subject to avoid the face and belly. The air blew out from the small side holes of the inner duct to avoid allowing the wind to directly hit the subjects (Fig. 1a). Both air conditioners had the highest and lowest temperature points at 42 °C and 11 °C, respectively, with a control accuracy of 0.5 °C at the outlet; the air conditioners for heating and cooling were set at their highest and lowest set-points, respectively. The airflow volume was set at 3.3 m^3^/min to further minimize the feeling of wind in the canopy by the subjects. Both air conditioners were placed outside the MRI scanner room with the air outlets connected to one end of a flexible duct that penetrated the wall of the MRI shield room and connected with the canopy at the other end. A damper system equipped with a timer-control function controlled the airflow so that the air from one air conditioner went into the canopy (Fig. 1b).

### Preliminary experiments

To determine several experimental parameters, preliminary experiments were conducted with the present authors as subjects. When a single conditioner worked continuously, it took approximately 30 min for the air temperature in the canopy to reach the hot or cold plateau from room temperature. The length of each heating or cooling phase during the fMRI experiment was adjusted based on the authors’ experience of being a subject in the experiments with different phase lengths; a 10-min period was the shortest time in which most of the authors (i.e., subjects) felt a shift in aerial temperature from uncomfortably cold to uncomfortably hot or vice versa. This time period is twice as short as that of a previous study using aerial thermal manipulation^36^ and twice as long as that of a study using a water-perfusion suit^34^. Under these conditions, the air temperature in the canopy ranged from 16–33 °C. The original plan was to administer a 40-min session to each subject twice but the preliminary experiments revealed that the subjects would be too exhausted to remain awake during the comfortable phase in the second session. In previous studies, each subject was administered only a single session^34, 36^, presumably for the same reason.

### Experimental procedures

First, the subjects adjusted their clothes until they felt comfortable in the ambient temperature of the scanner room (approximately 22 °C); most subjects took off the jacket or sweater they were wearing when they arrived. The thermal insulation of their clothing, estimated by ISO 9920^57^, was 0.65–0.85 clo (0.10–0.13 m^2^ °C/W). Strict control over clothing was avoided to more closely match the study environment with that of the subject’s daily life experience. Next, each subject lay on the bed of the MRI scanner with her/his head fixed in the head coil using a band and foam blocks. The fMRI session began with the heating phase for 18 subjects and with the cooling phase for 13 subjects.

### Objective measures

The air temperature in the canopy and the respiration rate of each subject were recorded using MRI-compatible transducers (TSD-202E and TSD-201, respectively; BIOPAC Systems Inc.; Goleta, California, USA). The temperature transducer was attached to the scanner bed close to the MRI head coil and the respiration transducer was attached around the upper abdomen of each subject. The signals from the transducers were amplified by constant current amplifiers (BIOPAC MP 100 system, BIOPAC Systems Inc.), sent to a laptop computer via fiber optic channels, and sampled at 1 Hz for digital recording.

The data were assessed with software for data acquisition and analysis from the BIOPAC MP 100 system (Acqknowledge, BIOPAC Systems Inc.). After the fMRI measurements, the data were visually inspected to exclude apparent noise. Due to technical reasons, temperature and respiration data were obtained from 28 and 23 subjects, respectively.

### Subjective measures

During the fMRI session, the subjects were instructed to independently rate their subjective thermal sensation and comfort levels every 30 sec by pressing a button on a 4-button MRI-compatible pad held in each hand. One pad was used for a 4-point scale to indicate thermal sensation (4: hot, 3: warm, 2: cool, and 1: cold) while the other pad for a 4-point scale to indicate thermal comfort (4: comfortable, 3: slightly comfortable, 2: slightly uncomfortable, and 1: uncomfortable); the button arrangements were counterbalanced across subjects. Each subject sequentially indicated his/her ratings in the order of thermal sensation and comfort following a cue (flashing light) presented inside the MRI gantry using a projector.

### fMRI measurement and preprocessing

All fMRI data were collected using a 3-Tesla Philips Achieva scanner equipped with an 8-channel head coil. The fMRI blood oxygenation level-dependent (BOLD) signal was measured with a fast field echo-echo planar imaging (FFE-EPI) sequence with the following characteristics: 64 × 64 matrix, TR = 2000 ms, TE = 30 ms, flip angle = 85°, FOV = 192 mm, slice thickness = 4.0 mm, gap = 0.5 mm, and 32 trans-axial slices per volume; 1200 functional volumes were acquired per run for each subject.

Image preprocessing was carried out using Statistical Parametric Mapping 12 (SPM12; Wellcome Department of Imaging Neuroscience; London, UK, http://www.fil.ion.ucl.ac.uk/spm/) and implemented on MATLAB (MathWorks, Natick MA, USA). All EPI images were (1) bias-field corrected with the bias field derived from the segmentation of the 600^th^ volume; (2) slice-time corrected with the first slice of each volume as reference slice; (3) unwarped and realigned to the first image in the time series to correct for head movement; (4) normalized to the Montreal Neurological Institute (MNI) reference space using the EPI template, deformation field derived from the mean EPI image, and resliced to a cubic voxel size of 2 mm^3^; and (5) smoothed with an isotropic Gaussian kernel with 5 mm full-width at half-maximum (FWHM). The choice of a relatively small smoothing kernel was based on a previous recommendation for the accurate mapping of small structures^58^.

### fMRI data analysis

Statistical fMRI analyses were performed at the individual and group levels using SPM12. The thermal sensation and comfort ratings were linearly interpolated into time series with an interval of 2.0 s (TR of the scan). These time series were specified as multiple regressors in the individual-level GLM without convolution of the hemodynamic response function. Due to the slow changes in these regressors, the standard discrete cosine transform (DCT) high-pass filter was not employed but the following three regressors were manually incorporated to regress out slow drifts: (1) a linear term, (2) a cosine term with whole scan duration (2400 s) as a half period, and (3) a cosine term with whole scan duration as a full period. The six realignment parameters estimated in the preprocessing procedure were also included in the GLM and the linear effects of the sensation and comfort regressors were estimated. At the group level, each of the individual estimates for sensation and comfort was analyzed with a one-sample *t*-test or two-sample *t*-test (i.e., for gender difference). The levels of statistical significance were set at a family-wise error (FWE)-corrected *p* value of <0.05 for cluster extent and an uncorrected *p* value of <0.005 for the cluster-forming threshold.

### Data availability

The MRI datasets generated and/or analyzed in the present study are not publicly available due to the risk of subject identification from the reconstructed images. However, the data are available from the corresponding author for reasonable requests.

## References

[1] Höppe P (1997). Aspects of human biometerology in past, present and future. International Journal of Biometeorology.

[2] de Dear RJ, Brager GS, Reardon J, Nicol F (1998). Developing an adaptive model of thermal comfort and preference/discussion. ASHRAE Transactions.

[3] Höppe P (2002). Different aspects of assessing indoor and outdoor thermal comfort. Energy and Buildings.

[4] Spagnolo J, de Dear R (2003). A field study of thermal comfort in outdoor and semi-outdoor environments in subtropical Sydney Australia. Building and Environment.

[5] de Dear RJ, Brager GS (2002). Thermal comfort in naturally ventilated buildings: revisions to ASHRAE Standard 55. Energy and Buildings.

[6] Nicol JF, Humphreys MA (2002). Adaptive thermal comfort and sustainable thermal standards for buildings. Energy and buildings.

[7] Mazon J (2014). The influence of thermal discomfort on the attention index of teenagers: an experimental evaluation. Int J Biometeorol.

[8] Seppänen OA, Fisk W (2006). Some quantitative relations between indoor environmental quality and work performance or health. Hvac&R Research.

[9] Vanos JK, Warland JS, Gillespie TJ, Kenny NA (2010). Review of the physiology of human thermal comfort while exercising in urban landscapes and implications for bioclimatic design. Int J Biometeorol.

[10] Brager GS, de Dear RJ (1998). Thermal adaptation in the built environment: a literature review. Energy and Buildings.

[11] Wu CF, Hsieh YF, Ou SJ (2015). Thermal adaptation methods of urban plaza users in Asia’s hot-humid regions: a Taiwan case study. Int J Environ Res Public Health.

[12] Knez I, Thorsson S (2008). Thermal, emotional and perceptual evaluations of a park: Cross-cultural and environmental attitude comparisons. Building and Environment.

[13] Kruger E, Drach P, Broede P (2016). Outdoor comfort study in Rio de Janeiro: site-related context effects on reported thermal sensation. Int J Biometeorol.

[14] Nikolopoulou M, Steemers K (2003). Thermal comfort and psychological adaptation as a guide for designing urban spaces. Energy and Buildings.

[15] Rutty M, Scott D (2015). Bioclimatic comfort and the thermal perceptions and preferences of beach tourists. Int J Biometeorol.

[16] Zhou X, Ouyang Q, Zhu Y, Feng C, Zhang X (2014). Experimental study of the influence of anticipated control on human thermal sensation and thermal comfort. Indoor Air.

[17] IJzerman H, Semin GR (2009). The thermometer of social relations mapping social proximity on temperature. Psychological Science.

[18] IJzerman H, Semin GR (2010). Temperature perceptions as a ground for social proximity. Journal of Experimental Social Psychology.

[19] Taufik D, Bolderdijk JW, Steg L (2014). Acting green elicits a literal warm glow. Nature Climate Change.

[20] Zhong C-B, Leonardelli GJ (2008). Cold and lonely does social exclusion literally feel cold?. Psychological Science.

[21] Keeling TP, Roesch EB, Clements-Croome D (2016). Cognitive appraisals affect both embodiment of thermal sensation and its mapping to thermal evaluation. Front Psychol.

[22] Becerra LR (1999). Human brain activation under controlled thermal stimulation and habituation to noxious heat: an fMRI study. Magnetic Resonance in Medicine.

[23] Casey KL, Minoshima S, Morrow TJ, Koeppe RA (1996). Comparison of human cerebral activation pattern during cutaneous warmth, heat pain, and deep cold pain. Journal of Neurophysiology.

[24] Craig AD, Reiman EM, Evans A, Bushnell MC (1996). Functional imaging of an illusion of pain. Nature.

[25] Davis KD, Kwan CL, Crawley AP, Mikulis DJ (1998). Functional MRI study of thalamic and cortical activations evoked by cutaneous heat, cold, and tactile stimuli. Journal of Neurophysiology.

[26] Moulton EA, Pendse G, Becerra LR, Borsook D (2012). BOLD responses in somatosensory cortices better reflect heat sensation than pain. J Neurosci.

[27] Tseng MT, Tseng WY, Chao CC, Lin HE, Hsieh ST (2010). Distinct and shared cerebral activations in processing innocuous versus noxious contact heat revealed by functional magnetic resonance imaging. Hum Brain Mapp.

[28] Craig AD, Chen K, Bandy D, Reiman EM (2000). Thermosensory activation of insular cortex. Nature Neuroscience.

[29] Hua LH, Strigo IA, Baxter LC, Johnson SC, Craig AD (2005). Anteroposterior somatotopy of innocuous cooling activation focus in human dorsal posterior insular cortex. Am J Physiol Regul Integr Comp Physiol.

[30] Peltz E (2011). Functional connectivity of the human insular cortex during noxious and innocuous thermal stimulation. Neuroimage.

[31] Davis KD, Pope GE, Crawley AP, Mikulis DJ (2004). Perceptual illusion of “paradoxical heat” engages the insular cortex. Journal of Neurophysiology.

[32] Olausson H (2005). Feelings of warmth correlate with neural activity in right anterior insular cortex. Neurosci Lett.

[33] Egan GF (2005). Cortical, thalamic, and hypothalamic responses to cooling and warming the skin in awake humans: a positron-emission tomography study. Proc Natl Acad Sci USA.

[34] Muzik O, Diwadkar VA (2016). *In vivo* correlates of thermoregulatory defense in humans: Temporal course of sub-cortical and cortical responses assessed with fMRI. Hum Brain Mapp.

[35] Nunneley SA (2002). Changes in regional cerebral metabolism during systemic hyperthermia in humans. Journal of Applied Physiology.

[36] Kanosue K (2002). Brain activation during whole body cooling in humans studied with functional magnetic resonance imaging. Neuroscience Letters.

[37] Rolls ET, Grabenhorst F, Parris BA (2008). Warm pleasant feelings in the brain. Neuroimage.

[38] Sung EJ (2007). Brain activation related to affective dimension during thermal stimulation in humans: a functional magnetic resonance imaging study. Int J Neurosci.

[39] Inagaki TK, Eisenberger NI (2013). Shared neural mechanisms underlying social warmth and physical warmth. Psychol Sci.

[40] Kang Y, Williams LE, Clark MS, Gray JR, Bargh JA (2011). Physical temperature effects on trust behavior: the role of insula. Soc Cogn Affect Neurosci.

[41] Kelly C (2012). A convergent functional architecture of the insula emerges across imaging modalities. Neuroimage.

[42] Mazzola L, Faillenot I, Barral FG, Mauguiere F, Peyron R (2012). Spatial segregation of somato-sensory and pain activations in the human operculo-insular cortex. Neuroimage.

[43] Klasen M, Kenworthy CA, Mathiak KA, Kircher TT, Mathiak K (2011). Supramodal representation of emotions. J Neurosci.

[44] Bastuji H, Frot M, Perchet C, Magnin M, Garcia-Larrea L (2016). Pain networks from the inside: Spatiotemporal analysis of brain responses leading from nociception to conscious perception. Hum Brain Mapp.

[45] Morimoto A, Murakami N (1985). [14C] deoxyglucose incorporation into rat brain regions during hypothalamic or peripheral thermal stimulation. American Journal of Physiology-Regulatory, Integrative and Comparative Physiology.

[46] Morimoto A (1986). Changes in [14 C] deoxyglucose incorporation into rat brain regions during heat-seeking behavior in the cold environment. Physiology and Behavior.

[47] Nakagawa H, Matsumura T, Suzuki K, Ninomiya C, Ishiwata T (2016). Changes of brain monoamine levels and physiological indexes during heat acclimation in rats. J Therm Biol.

[48] Balleine BW, Delgado MR, Hikosaka O (2007). The role of the dorsal striatum in reward and decision-making. J Neurosci.

[49] Devan BD, Hong NS, McDonald RJ (2011). Parallel associative processing in the dorsal striatum: segregation of stimulus-response and cognitive control subregions. Neurobiol Learn Mem.

[50] Craig AD (2002). How do you feel? Interoception: the sense of the physiological condition of the body. Nature Reviews Neuroscience.

[51] Craig, A. D. How do you feel—now? the anterior insula and human awareness. *Nature Reviews Neuroscience* **10** (2009). 10.1038/nrn2555 19096369

[52] Sugiura M, Shah NJ, Zilles K, Fink GR (2005). Cortical representations of personally familiar objects and places: functional organization of the human posterior cingulate cortex. J Cogn Neurosci.

[53] Lei LG, Zhang YQ, Zhao ZQ (2004). Pain-related aversion and Fos expression in the central nervous system in rats. Neuroreport.

[54] Farrer C (2003). Modulating the experience of agency: a positron emission tomography study. NeuroImage.

[55] Hsu M, Anen C, Quartz SR (2008). The right and the good: distributive justice and neural encoding of equity and efficiency. Science.

[56] Nahab FB (2011). The neural processes underlying self-agency. Cereb Cortex.

[57] ISO. ISO 9920: Ergonomics of the thermal environment: estimation of thermal insulation and water vapour resistance of a clothing ensemble. (Geneva, 2007).

[58] Sacchet MD, Knutson B (2013). Spatial smoothing systematically biases the localization of reward-related brain activity. Neuroimage.

